# Long-term outcomes of transapical-transcatheter aortic valve replacement

**DOI:** 10.1007/s11748-024-02095-x

**Published:** 2024-10-23

**Authors:** Koichi Maeda, Kazuo Shimamura, Isamu Mizote, Daisuke Nakamura, Kizuku Yamashita, Ai Kawamura, Daisuke Yoshioka, Yasushi Sakata, Shigeru Miyagawa

**Affiliations:** 1 Department of Cardiovascular Surgery, Osaka University Graduate School of Medicine, 2-2 Yamadaoka, Suita, 565-0871 Osaka Japan; 2 Department of Cardiology, Osaka University Graduate School of Medicine, 2-2 Yamadaoka, Suita, 565-0871 Osaka Japan

**Keywords:** Transapical-transcatheter aortic valve replacement, Aortic stenosis, Alternative approach

## Abstract

**Objective:**

Transapical-transcatheter aortic valve replacement is one of the main interventions indicated for patients where access via peripheral vessels is challenging. However, there have been no reports on the long-term outcomes of this intervention. Here, we report the long-term outcomes of this intervention.

**Methods:**

Among 178 patients who underwent transapical-transcatheter aortic valve replacement between October 2009 and July 2023, 173 patients who underwent this intervention for native aortic stenosis were included in this study, and early and long-term results were evaluated.

**Results:**

The mean age was 82.4 ± 6.4 years, 52.6% were women, mean body area was 1.46 ± 0.17 m^2^, and the Society of Thoracic Surgeons Predicted Risk of Mortality was 11.2 ± 9.9%. In-hospital mortality was observed in three patients (1.7%). Mean follow-up duration was 4.3 ± 2.8 years, and the survival rates at 1-, 3-, 5-, and 8-years were 84.9%, 67.1%, 47.0%, and 22.1%, respectively. Freedom from cardiovascular mortality at 1, 3, 5, and 8-years was 92.9%, 86.1%, 75.8%, and 53.5%, respectively. The freedom from disabling stroke rates at 1, 3, 5, and 8-years were 95.0%, 92.4%, 92.4%, and 90.8%, respectively. Multivariate analysis revealed that male (Hazard Ratio 1.85, 95%Confidence Interval 1.27−2.70, p = 0.0012) and hemodialysis (Hazard Ratio 1.64, 95%Confidence Interval 1.00−2.67, p = 0.049) were significant poor prognosis factors.

**Conclusions:**

Long-term outcomes of transapical-transcatheter aortic valve replacement were satisfactory. Despite the variety of available approaches, the role of transapical-transcatheter aortic valve replacement, which has low vascular impact, has not been completely lost.

**Supplementary Information:**

The online version contains supplementary material available at 10.1007/s11748-024-02095-x.

## Introduction

Transcatheter aortic valve replacement (TAVR), a safe and effective treatment for severe aortic stenosis (AS), has evolved rapidly over the past decade from its initial indication for inoperable or high-risk patients to its current indication for low-risk patients. Transfemoral transcatheter aortic valve replacement (TF-TAVR) is the most common because it is less invasive, whereas transapical-transcatheter aortic valve replacement (TA-TAVR) is one of the central approaches of TAVR and is indicated for patients in whom it is difficult to approach from peripheral vessels. However, with advances in catheters, the number of cases in which TF-TAVR can be performed, even in Japanese patients with small body size, has increased, while the number of TA-TAVR cases has significantly decreased. Recently, TAVR for dialysis patients was approved in Japan. In general, dialysis patients often have peripheral vessel diseases and require central access, which has renewed the interest in TA-TAVR. There have been no reports on the long-term outcomes of TA-TAVR, and herein, we report them.

## Subjects

Among 178 patients undergoing TA-TAVR at our institute between October 2009 and July 2023, five patients undergoing valve-in-valve therapy were excluded from this study. A total of 173 patients were enrolled in this study.

## Methods

### Procedure

The left fifth or sixth intercostal spaces were opened. The puncture site was between the left anterior descending artery and the diagonal branch but was not a so-called “dimple” because of thinner wall thickness. A double purse-string suture with eight round felts and 3–0 Prolene (SH) was placed (Supplementary Fig. 1). The needle was inserted firmly and deeply and care was taken not to penetrate the myocardium. To remove the sheath, we tied the suture gently after lowering the blood pressure sufficiently using rapid pacing.

### Data source

SAPIEN (Edwards Lifesciences, Irvine, California) and ACURATE (Symetis SA, Ecublens, Switzerland) were used in the clinical studies, while SAPIEN XT and SAPIEN 3 (Edwards Lifesciences) were covered by insurance. Aspirin was administered to all patients after TA-TAVR. However, additional antiplatelet and/or anticoagulation agents were administered after considering patient background and medications used by the patient before TAVR.

Patients underwent follow-up examinations, including transthoracic echocardiography, at the time of the procedure, at discharge from the hospital or at postoperative day-7, at 30 days, 6 months, and 12 months post-operatively, and then annually afterward. In case of missing questionnaires or the occurrence of adverse events, telephonic and personal consultations were conducted.

Cardiovascular mortality, stroke, and bleeding were defined according to the Valve Academic Research Consortium 3 criteria [1]. The primary endpoint of this study was all-cause mortality, and the secondary endpoints were cardiovascular mortality, disabling stroke, and structural valve deterioration (SVD). The therapies for these patients were discussed at multidisciplinary meetings attended by cardiac surgeons, cardiologists, and anesthesiologists. This study was approved by the Institutional Review Board of Osaka University, Japan and all patients provided written informed consent (Approval Number: 16105–3).

### Statistical analysis

Continuous variables are presented as mean ± standard deviation or median (interquartile range [IQR]), and categorical variables as frequencies (%). Group differences were assessed using 1-way analysis of variance or the Kruskal–Wallis test, depending on their distributions. Overall survival, freedom from cardiovascular mortality, freedom from disabling stroke, and freedom from SVD analyses were performed using the Kaplan–Meier method, with patient data censored as of the last date the patient was known to be alive. Logistic regression analysis was performed to examine predictors of late mortality. Two patients with severe paravalvular leakage due to delayed valve migration at 13 days and 6 months after TAVR were excluded from the analysis of valve dysfunction because of the potential for technical failure (implantation in a lower position). Statistical analyses were performed using statistical analysis software, JMP® Pro, Version 17.1.0 (SAS Institute, Inc., Cary, NC, USA).

## Results

The mean age of our patient cohort was 82.4 ± 6.4 years and 52.6% were women. The mean body surface area (BSA) and body mass indices were 1.46 ± 0.17 m^2^ and 21.8 ± 3.3 kg/m^2^, respectively. Thirty-five patients (20.2%) had a history of cardiac surgery and 26 patients (15.0%) underwent hemodialysis. Peripheral vessel disease was found in 48.6% and the mean Society of Thoracic Surgeons (STS) Predicted Risk of Mortality (PROM) was 11.2 ± 9.9%. The other baseline characteristics are shown in Table 1.

**Table 1:** Baseline patient characteristics

Patients, n	173
Demographics and physical status	
Age, years (mean ± SD)	82.4 ± 6.4
< 65, no. of pts (%)	2 (1.2)
65–74, no. of pts (%)	18 (10.4)
75–79, no. of pts (%)	30 (17.3)
80–84, no. of pts (%)	58 (33.5)
85 ≤ , no. of pts (%)	65 (37.5)
Sex (Female), no. of pts (%)	91 (52.6)
BSA, kg/m^2^ (mean ± SD)	1.46 ± 0.17
BMI (mean ± SD)	21.8 ± 3.3
NYHA III or IV, no. of pts (%)	83 (48.0)
CAD, no. of pts (%)	73 (42.2)
Atrial fibrillation/ atrial flutter, no. of pts (%)	22 (12.7)
COPD (moderate or severe), no. of pts (%)	38 (22.9)
Hypertension, no. of pts (%)	160 (92.5)
DM, no. of pts (%)	42 (24.3)
Insulin-dependent DM, no. of pts (%)	10 (5.8)
Peripheral vessel disease, no. of pts (%)	84 (48.6)
Previous cardiac surgery, no. of pts (%)	35 (20.2)
Hemodialysis, no. of pts (%)	26 (15.0)
Albumin, g/dL (mean ± SD)	3.7 ± 0.4
STS-PROM, % (mean ± SD)	11.2 ± 9.9
< 4, no. of pts (%)	15 (8.7)
4–8, no. of pts (%)	62 (35.8)
8 ≤ , no. of pts (%)	96 (55.5)
Echocardiographic findings	
Aortic regurgitation grade	
None or trivial, no. of pts (%)	43 (24.9)
Mild, no. of pts (%)	99 (57.2)
Moderate, no. of pts (%)	28 (16.2)
Severe, no. of pts (%)	3 (1.7)
Mitral regurgitation ≥ moderate, no. of pts (%)	
None or trivial, no. of pts (%)	70 (40.5)
Mild, no. of pts (%)	93 (53.8)
Moderate, no. of pts (%)	9 (5.2)
Severe, no. of pts (%)	1 (0.5)
LVEF at baseline, % (mean ± SD)	61.7 ± 13.1
Aortic valve mean gradient at baseline, mmHg (mean ± SD)	48.1 ± 16.1
Aortic valve area at baseline, cm^2^ (mean ± SD)	0.72 ± 0.16
Indexes Aortic valve area at baseline, cm^2^/m^2^ (mean ± SD)	0.49 ± 0.11

*no. of pts* number of patients, *SD* standard deviation, *BSA* body surface area, *BMI* body mass index, *NYHA* New York Heart Association, *CAD* coronary artery disease, *COPD* chronic obstructive pulmonary disease, *DM* diabetes mellitus, *STS-PROM* Society of Thoracic Surgeons Predicted Risk of Mortality, *LVEF* left ventricular ejection fraction

### In-hospital outcomes

Table 2 lists in-hospital outcomes. SAPIEN (n = 26, 15.0%), SAPIEN XT (n = 108, 62.4%), SAPIEN 3 (n = 30, 17.3%), and ACURATE (n = 9, 5.2%) were implanted. Concomitant procedures included coronary artery bypass grafting in five patients and tricuspid annuloplasty in one patient. Median operation time and median blood loss were 102 min and 350 ml, respectively. In 167 cases, except for the concomitant procedures, the operating time tended to decrease with the number of cases (Spearman *r* = 0.08, p = 0.0002) (Supplementary Fig. 2). Further, these two surgical parameters were significantly improved in patients using SAPIEN 3 compared to those using non-SAPIEN 3 (operation time: 106 vs 81 min; p = 0.0015, blood loss: 380 vs 150 mL; p = 0.0053). Sixteen patients (9.8%) required new permanent pacemakers. The in-hospital deaths of three patients (1.7%) were observed: one from annulus rupture, one from perioperative myocardial infarction, and one from acute heart failure due to massive mitral regurgitation resulting from papillary muscle injury. Disabling stroke was found in five patients (2.9%). Of the total patients, 87.9% were discharged home and the median postoperative hospital stay was 10 days.

**Table 2:** In-hospital outcomes

Patients, n	173
Transcatheter heart valve	
SAPIEN, no. of pts (%)	26 (15.0)
SAPIEN XT, no. of pts (%)	108 (62.4)
SAPIEN 3, no. of pts (%)	30 (17.3)
ACURATE, no. of pts (%)	9 (5.2)
Concomitant procedure, no. of pts (%)	6 (3.5)
CABG, no. of pts (%)	5 (2.9)
TAP, no. of pts (%)	1 (0.6)
THV size	
SAPIEN (n = 26)	
20 mm, no. of pts (%)	0 (0)
23 mm, no. of pts (%)	15 (57.7)
26 mm, no. of pts (%)	11 (42.3)
29 mm, no. of pts (%)	0 (0)
SAPIEN XT (n = 108)	
20 mm, no. of pts (%)	2 (1.9)
23 mm, no. of pts (%)	56 (51.9)
26 mm, no. of pts (%)	47 (43.5)
29 mm, no. of pts (%)	3 (2.8)
SAPIEN 3 (n = 30)	
20 mm, no. of pts (%)	0 (0)
23 mm, no. of pts (%)	12 (40.0)
26 mm, no. of pts (%)	14 (46.7)
29 mm, no. of pts (%)	4 (13.3)
ACURATE (n = 9)	
23 mm, no. of pts (%)	2 (22.2)
25 mm, no. of pts (%)	0 (0)
27 mm, no. of pts (%)	7 (77.8)
Operating time, min (median)	102
Blood loss, ml (median)	350
Requirement of permanent pacemaker^a^, no. of pts (%)	16 (9.8)
Disabling stroke, no. of pts (%)	5 (2.9)
postoperative hospital stay, days (median)	10
In-hospital mortality	3 (1.7)
Discharge to home, no. of pts (%)	152 (87.9)

*no. of pts* number of patients, *SD* standard deviation, *CABG* coronary artery bypass grafting, *TAP* tricuspid annular plasty, *THV* transcatheter heart valve

^a^Population of 164 patients, excluding nine patients with pre-existing pacemakers

### Long-term outcomes

**Fig. 1:**
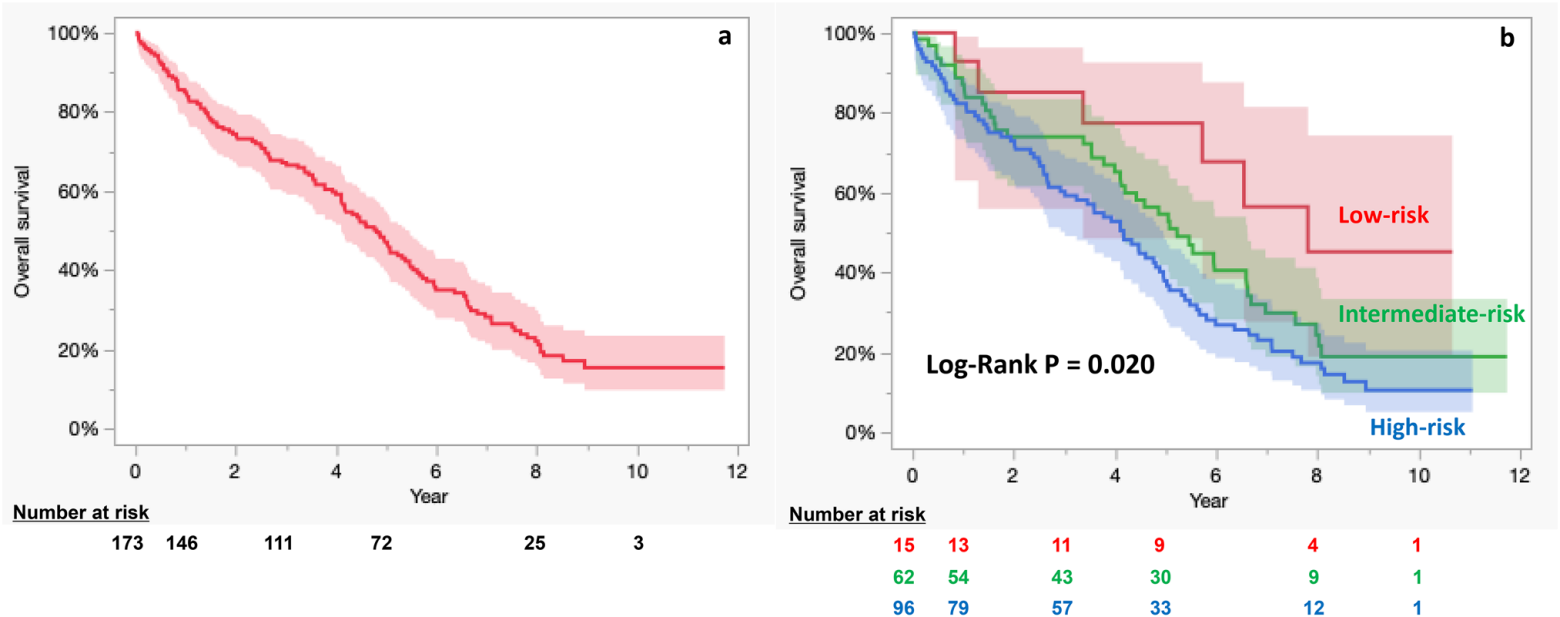
Kaplan–Meier analysis of overall survival (**a**) and overall survival associated with the STS-PROM classification (**b**). STS-PROM, Society of Thoracic Surgeons Predicted Risk of Mortality

**Fig. 2:**
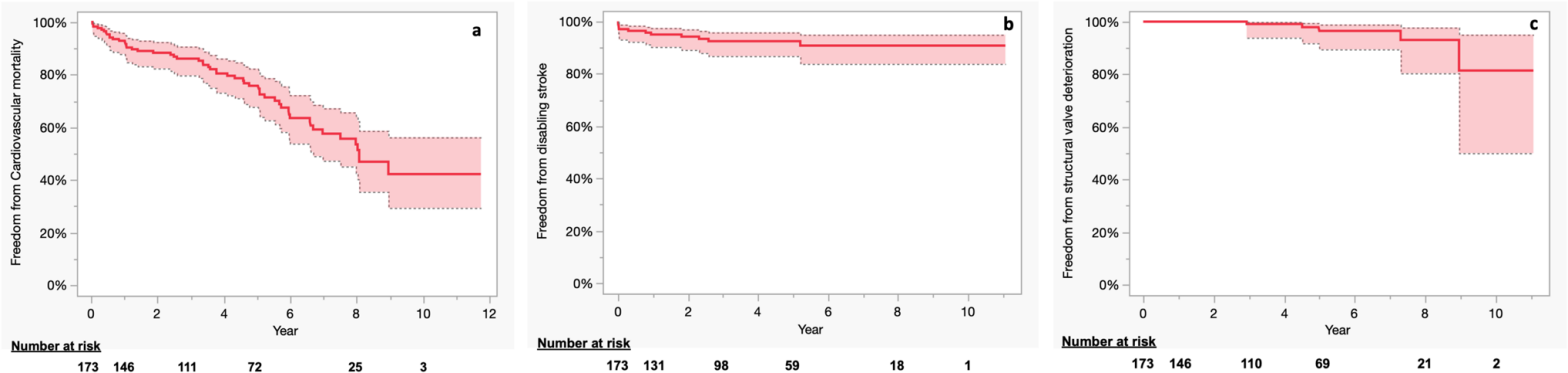
Kaplan–Meier analysis of freedom from cardiovascular mortality (**a**), freedom from disabling stroke (**b**), and freedom from structural valve deterioration (**c**)

The mean duration for follow-up was 4.3 ± 2.8 years (total follow-up duration: 748 person-years) and the follow-up rate was 92.5%. There were 46 deaths, resulting in an incidence rate of 6.1 deaths /100 person-years. The survival rates at 1, 3, 5, and 8-years were 84.9%, 67.1%, 47.0%, and 22.1%, respectively (Fig. 1a). The overall survival rate of non-hemodialysis patients compared very favorably with that of hemodialysis patients (Supplementary Fig. 3). Furthermore, the survival rates were associated with the STS-PROM classification (P = 0.020); the survival rates at 1, 3, 5, and 8 years were 92.9%, 85.1%, 77.4%, and 45.1% in the low-risk group (STS-PROM < 4%), respectively; 86.4%, 74.0%, 54.6%, and 24.4%, respectively, in the intermediate-risk group (STS-PROM 4–8%); and 82.8%, 60.3%, 37.8%, and 17.4%, respectively, in the high-risk group (STS-PROM > 8%), respectively (Fig. 1b). The rates of freedom from cardiovascular mortality at 1, 3, 5, and 8-years were 92.9%, 86.1%, 75.8%, and 53.5%, respectively (Fig. 2a). Furthermore, freedom from disabling stroke rates at 1, 3, 5, and 8-years were 95.0%, 92.4%, 92.4%, and 90.8%, respectively (Fig. 2b). Non-hemodialysis patients did not differ from hemodialysis patients in terms of freedom from cardiovascular events (Supplementary Fig. 4a) but were superior in terms of freedom from disabling stroke events (Supplementary Fig. 4b).

Multivariate analysis revealed that the male sex (HR 1.85, 95%CI 1.27−2.70, p = 0.0012) and hemodialysis (HR 1.64, 95%CI 1.00−2.67, p = 0.049) were significantly associated with all-cause mortality (Table 3). SVD was observed in five patients, one of whom was on hemodialysis. A higher rate of freedom from SVD was noted among the non-hemodialysis patients (Supplementary Fig. 4c). The rates of freedom from SVD at 1, 3, 5, and 8-years were 100%, 99.1%, 96.5%, and 93.0%, respectively (Fig. 2c).

**Table 3:** Uni- and multivariate analysis for all-cause mortality

	Univariate analysis		Multivariate analysis	
	HR (95% CI)	P value	HR (95% CI)	P value
Age (per 1 year increment)	1.02 (0.99–1.05)	0.21		
Sex (male)	1.80 (1.06–3.04)	0.030	1.85 (1.27–2.70)	0.0012
BSA (per 1 m^2^ increment)	1.17 (0.24–5.74)	0.85		
Hemodialysis	1.69 (0.95–3.00)	0.084	1.64 (1.00–2.67)	0.049
STS-PROM (per 1% increment)	1.02 (0.99–1.05)	0.072	1.01 (1.00–1.03)	0.12
Atrial fibrillation	0.90 (0.50–1.60)	0.71		
Peripheral vessel disease	0.94 (0.62–1.42)	0.77		
COPD ≥ moderate	0.79 (0.50–1.26)	0.31		
Insulin-dependent DM	1.59 (0.67–3.75)	0.31		
NYHA ≥ III	1.02 (0.99–1.05)	0.21		
LVEF (per 1% increment)	0.99 (0.98–1.01)	0.57	0.99 (0.98–1.01)	0.26
Albumin (per 1 mg/dl increment)	0.80 (0.49–1.30)	0.36	0.72 (0.47–1.14)	0.16

*CI* confidence interval, *HR* hazard ratio, *BSA* body surface area, *STS-PROM* Society of Thoracic Surgeons-Predicted Risk of Mortality, *COPD* chronic obstructive pulmonary disease, *DM* diabetes mellitus, *NYHA* New York Heart Association, *LVEF* left ventricular ejection fraction

**Fig. 3:**
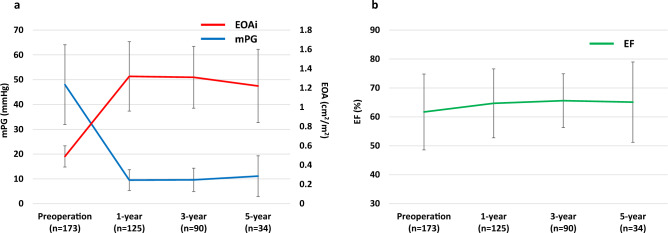
Hemodynamic data at follow-up Showing mean pressure gradient and indexed effective orifice (**a**), as well as left ventricular ejection fraction (**b**)

Hemodynamic data at follow-up are shown in Fig. 3. The mean pressure gradient and indexed effective orifice area were well-maintained during the follow-up period (Fig. 3a). Furthermore, left ventricular ejection fraction did not change during the follow-up period (Fig. 3b).

## Discussion

TAVR has evolved rapidly over the past decade from its initial introduction in high-risk or inoperable patients to its use in low-risk populations. Data regarding the mid- and long-term clinical outcomes of TAVR continue to be scarce and challenging to obtain, owing to the small number of patients operated on in the early experience and surviving for a long time. The survival data reported in our study showed good early- and mid-term outcomes, followed by a reduction at the 5- and 8-years follow-up (47.0% at the 5-years follow-up and 22.1% at the 8-years follow-up). However, the main finding of this single-center experience was that the long-term results of TA-TAVR were quite satisfactory; in particular, it is worth noting that in the very high-risk patient group, freedom from cardiovascular death showed a satisfactory value of 52.9% at 8 years post-procedure. Further, freedom from disabling stroke was considered satisfactory at 92.4% at 8 years post-procedure. These results are in good agreement with those reported by previous studies on TA-TAVRs [2–8]. We hypothesize that these outcomes may reflect the proportion of our TA experience (i.e., 40% of the TAVR procedures at the beginning were TA-TAVR procedures) that allowed us to overcome the learning curve, as shown in Supplementary Fig. 2. Similar to our results, Murashita et al. reported that, despite differences in baseline patient risk, the surgical and long-term mortality rates for TA-TAVR and TF-TAVR were similar, supporting the hypothesis that access does not affect treatment-related mortality [5]. Although STS-PROM are generally predictive of early (30-day) outcomes, in a report examining the relationship between higher STS-PROM and observed mortality and morbidity in the TF-TAVR and non-TF-TAVR groups, patients with higher STS PROM (8% or higher) in non-TF-TAVR groups was associated with higher 1-year mortality. In our analysis, when the patients undergoing TA-TAVR were further stratified into low-, intermediate-, and high-risk groups according to STS-PROM, we observed a correlation between STS-PROM and prognosis. This may be because the prognosis of patients who are candidates for TA-TAVR is strongly associated with comorbidities, such as peripheral vessel disease.

The present study was not a comparison with TF-TAVR, but a single-arm study. TA-TAVR is generally considered to have poorer results than TF-TAVR [2, 9, 10], and this is generally attributed to the clinical background of the TA-TAVR patients. In fact, there are reports of no difference between the two groups in matched studies [8, 11], but there are also reports of TF-TAVR being superior even after such matching [12, 13], and this issue remains controversial in the absence of prospective studies. In particular, the PARTNER-I substudy showed that when TA-TAVR was matched with TF-TAVR, TA-TAVR had a similar stroke risk and less aortic regurgitation than TF-TAVR in patients with vascular disease, but with a higher likelihood of periprocedural adverse events and longer recovery; they concluded that the TF-first access strategy is recommended if anatomically feasible [4]. Myocardial injury following TA-TAVR is a major concern. Amold et al. measured the creatine kinase-myocardial band and troponin T levels after TAVR procedures to assess myocardial injury before and after TAVR in non-transfemoral candidates. They observed myocardial injury in all such patients; however, TA-TAVR was associated with significantly greater myocardial injury than the transaortic/direct aortic approach. Furthermore, a higher degree of myocardial injury was observed to be associated with a reduced improvement in left ventricular function and reduced early and mid-term survival [14]. However, there are a few reports that TA-TAVR has no effect on LV myocardial injury, but these are retrospective studies where patient selection bias cannot be ruled out [6, 7]. The results of the present study showed that the ejection fraction did not change during follow-up, and this may indicate only minor myocardial damage at the approach site.

The development of new alternative approaches, including the trans-subclavian, direct aortic, and trans-carotid approaches, has led to a steady decline in the overall number of TA-TAVRs [15]. However, there are still a certain number of patients, such as hemodialysis patients, for whom peripheral access (transfemoral, transsubclavian, transaxillary, and transcarotid) is challenging and for whom central access (including transapical and transaortic) appears to have advantages. Among the alternative approaches, Kaneko et al. reported that all-cause mortality was significantly lower in the peripheral versus central access group at the in-hospital and 1-year post-procedure stages, but stroke rates were higher [16]. These results persisted after a 1-year adjustment. Therefore, the authors of that study concluded that an accurate prognosis of risk is mandatory for patient counseling and decision-making by the heart surgery team regarding alternative access to TAVR. Our heart team concurred. We also tended to choose the transaortic, transapical, or transsubclavian approaches (in that order) as alternatives approaches, if feasible. Recent approval of the carotid approach may change this policy. However, we believe that the role of TA-TAVR, which has an infinitely low vascular impact, is not completely lost.

### Study limitations

The main limitations of this study are as follows. First, it had a non-randomized retrospective single-center design, and a small sample size. Additional limitations included: (1) data on clinical and echocardiographic follow-ups was not available for all patients, and (2) considerable technical advancement in transcatheter valves occurred throughout the study period and is ongoing. Finally, the low-risk trial only examined the transfemoral approach [17]. Although an alternative approach cohort existed as a substudy, it was not shown to be a useful alternative. The results of the present study were more favorable among low-risk patients. Importantly, as the Japanese guidelines state that unfavorable femoral access favors SAVR over TAVR, these results do not mean that alternative TAVR is actively recommended, even for low-risk patients.

## Conclusions

Long-term TA-TAVR results were satisfactory. Although a variety of surgical approaches are available, there are still a small number of cases where the TA-TAVR surgical approach is better and where the use of TA-TAVR should not be allowed to decline in surgically relevant cases.

## Supplementary Information

Below is the link to the electronic supplementary material.

Supplementary file1 (PPTX 280 KB)

Supplementary file2 (PPTX 83 KB)

Supplementary file3 (PPTX 78 KB)

Supplementary file4 (PPTX 342 KB)

## Data Availability

The raw data that support the findings of this study are available from the corresponding author, upon reasonable request.
