# Supplementary figures and images for: Long-term outcomes of transapical-transcatheter aortic valve replacement
Source: Gen Thorac Cardiovasc Surg. 2024 Oct 23;73(7):472–8. doi: 10.1007/s11748-024-02095-x (PMC12174220; doi:10.1007/s11748-024-02095-x)

## Slide 1
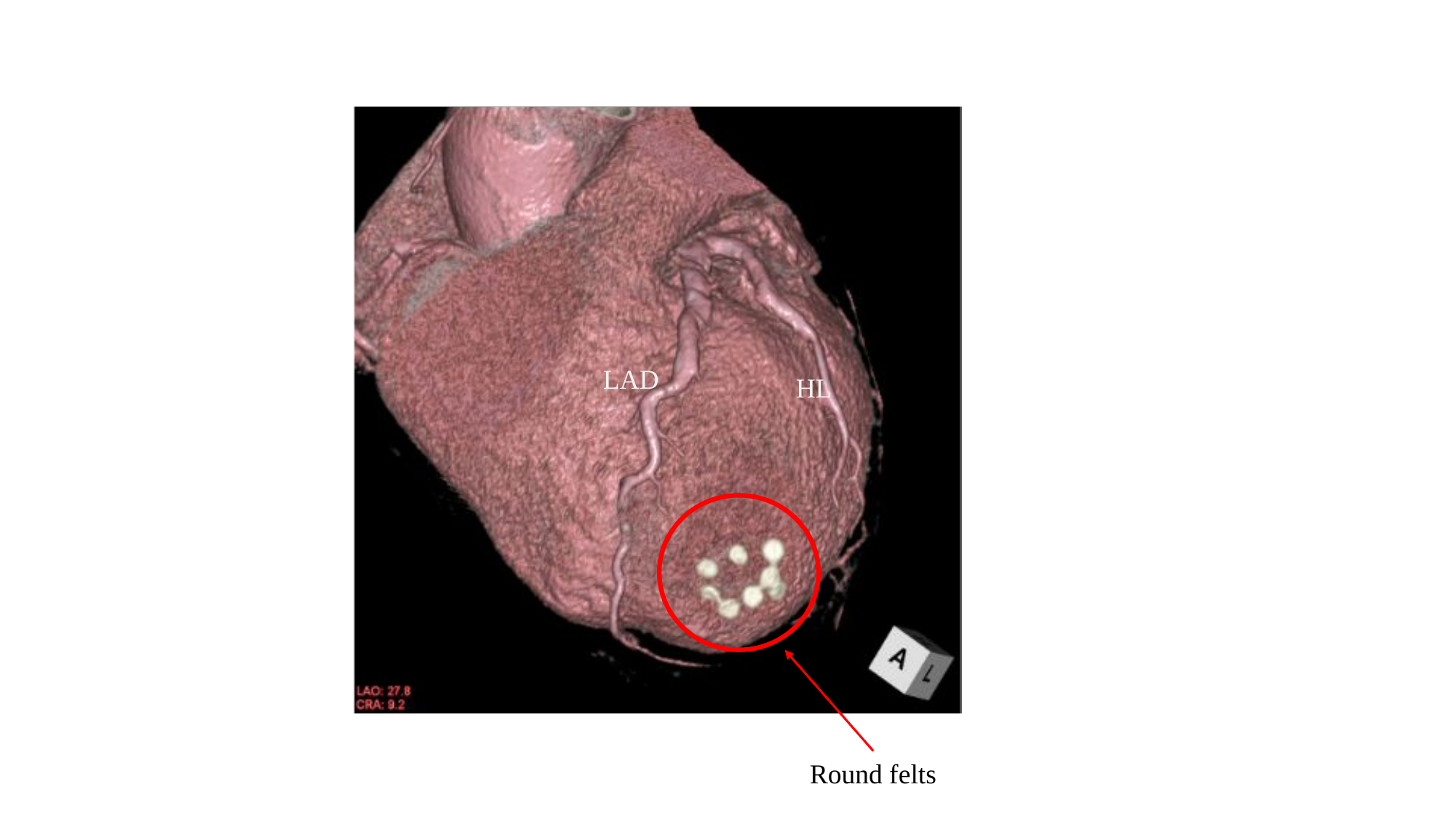

LAD
HL
Round felts

Supplement: Supplementary file 1 — Supplementary file1 (PPTX 280 KB) [file 11748_2024_2095_MOESM1_ESM.pptx]

## Slide 1
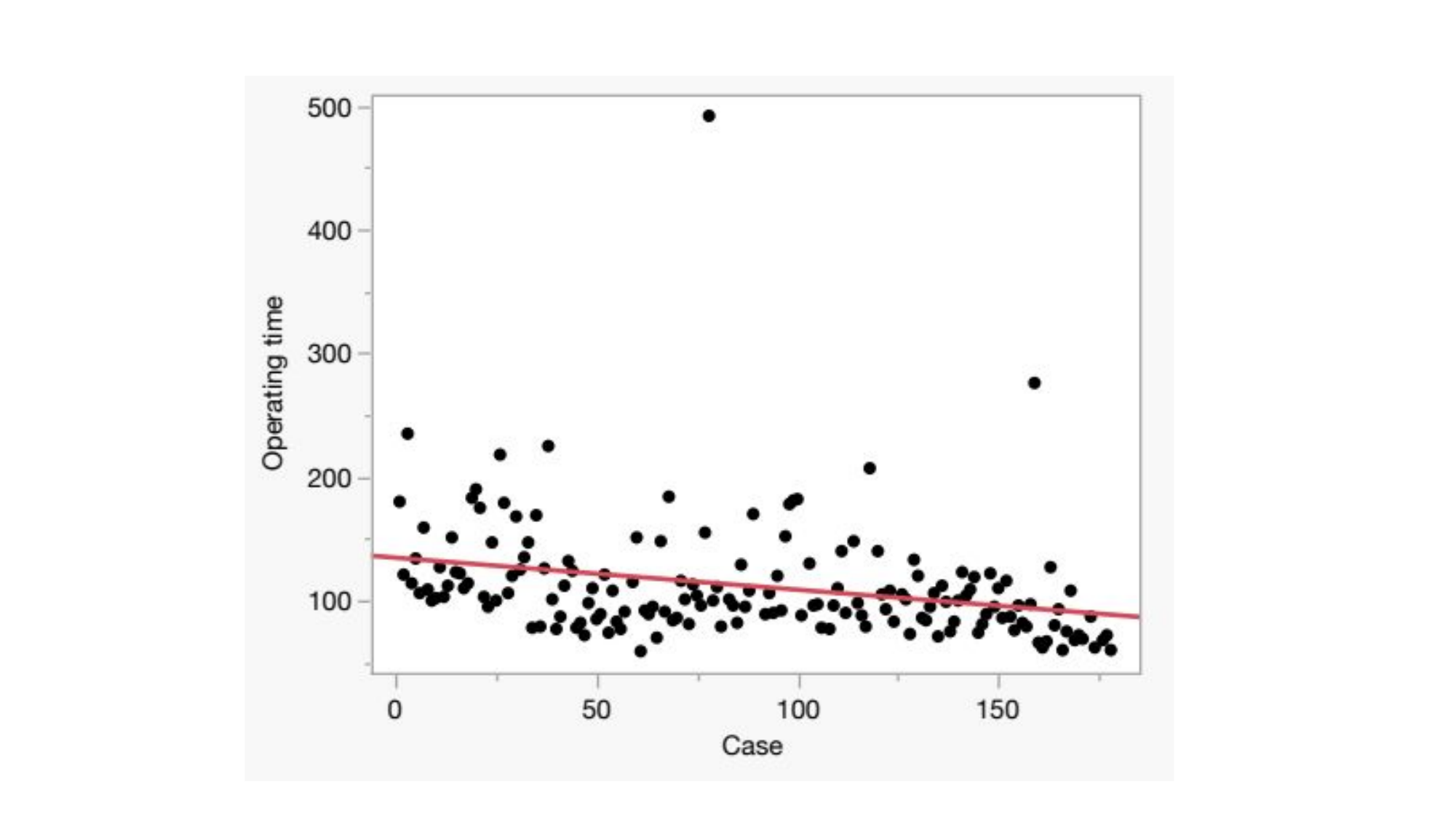

Supplement: Supplementary file 2 — Supplementary file2 (PPTX 83 KB) [file 11748_2024_2095_MOESM2_ESM.pptx]

## Slide 1
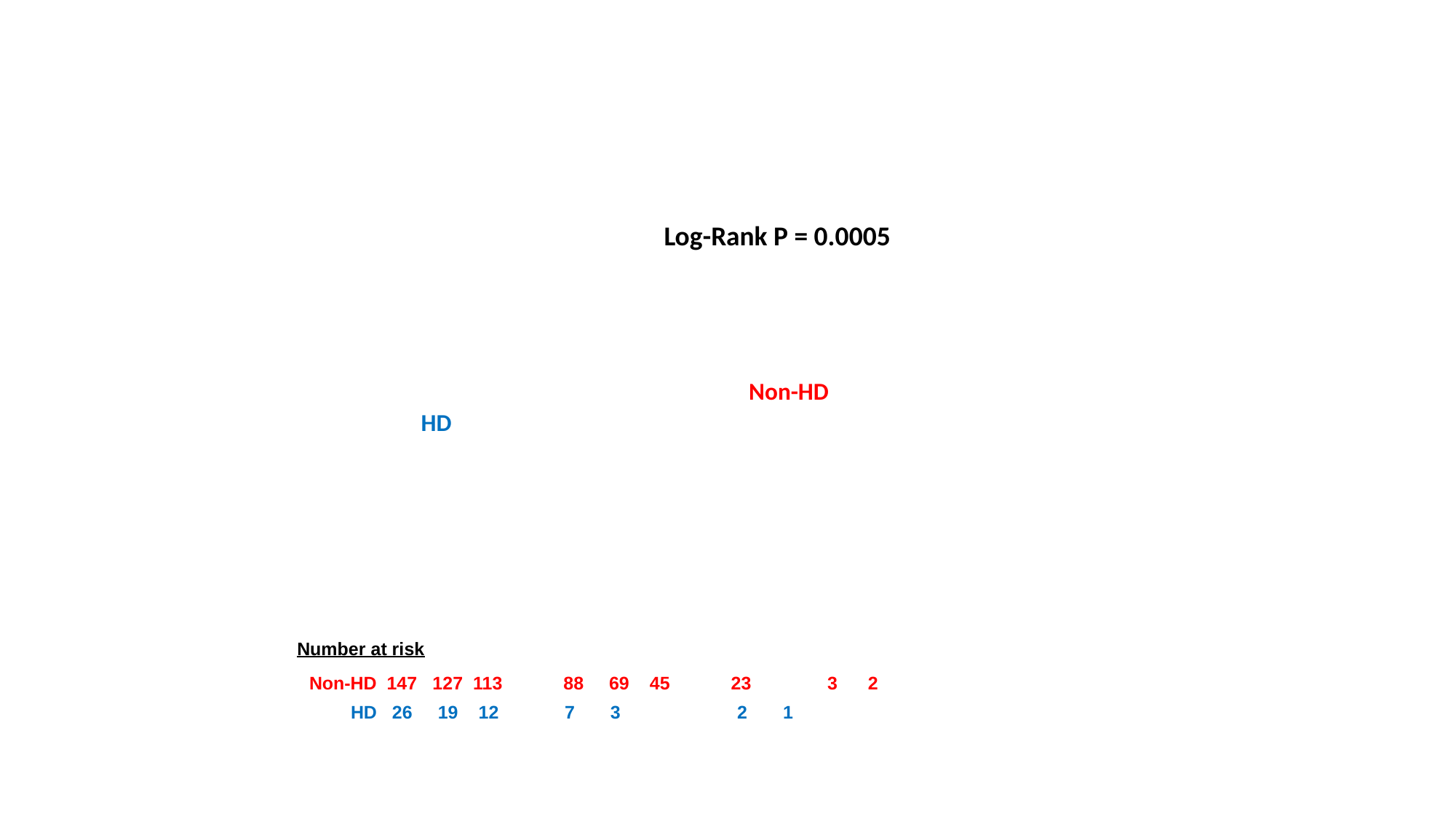

Log-Rank P = 0.0005
Non-HD
HD
Number at risk
Non-HD 147 127 113 88 69 45 23 3 2
 HD 26 19 12 7 3 2 1

Supplement: Supplementary file 3 — Supplementary file3 (PPTX 78 KB) [file 11748_2024_2095_MOESM3_ESM.pptx]

## Slide 1
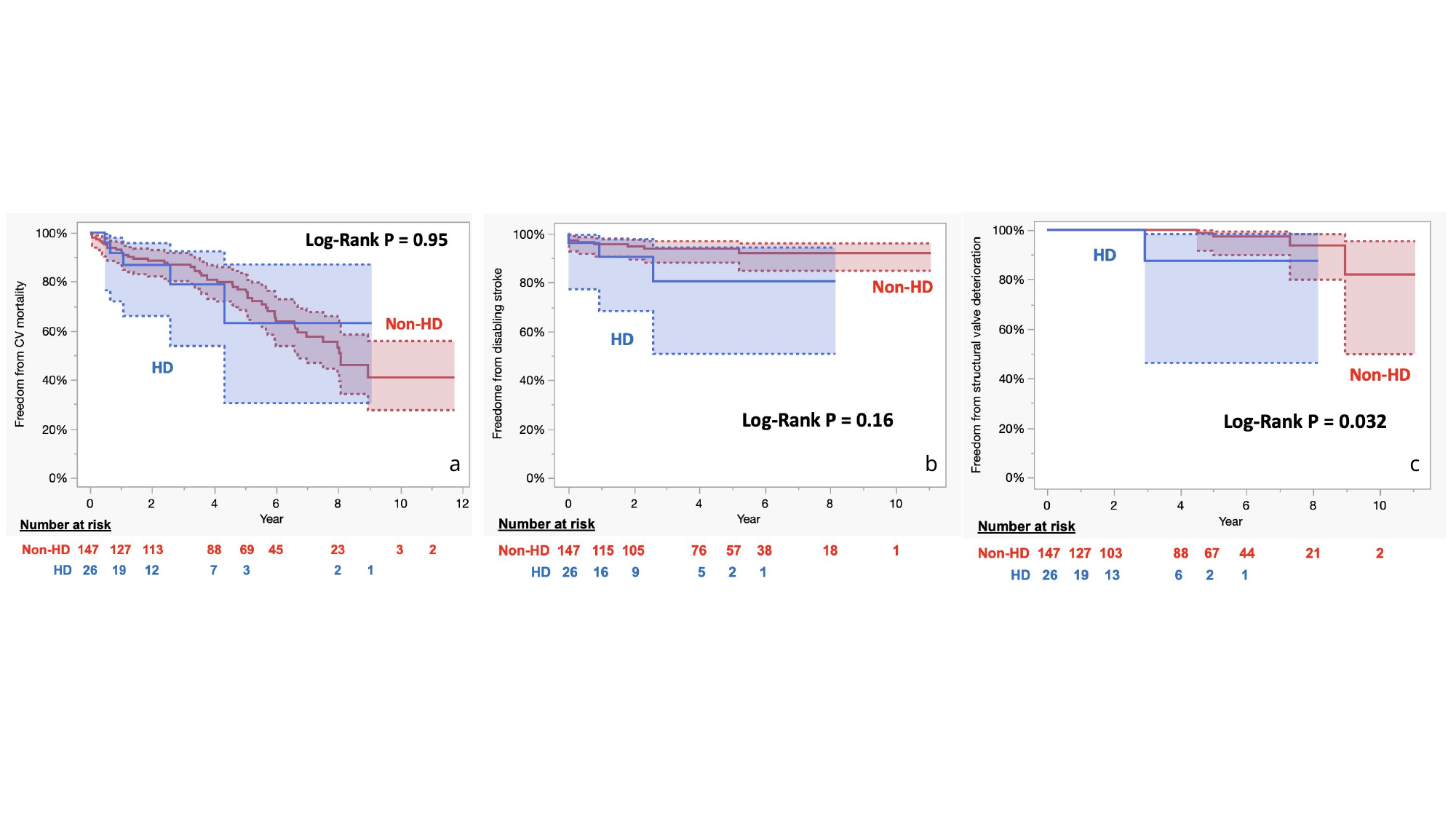

b
c
a

Supplement: Supplementary file 4 — Supplementary file4 (PPTX 342 KB) [file 11748_2024_2095_MOESM4_ESM.pptx]
